# A High-Sensitivity SPR Refractive Index Sensor Based on No-Core Fiber with Ag-Cu Composite Films

**DOI:** 10.3390/s21217000

**Published:** 2021-10-22

**Authors:** Yuhui Feng, Hongyu Li, Shuguang Li, Yundong Liu, Xiaojian Meng

**Affiliations:** State Key Laboratory of Metastable Materials Science & Technology and Key Laboratory for Microstructural Material Physics of Hebei Province, School of Science, Yanshan University, Qinhuangdao 066004, China; fengyuhui@stumail.ysu.edu.cn (Y.F.); hongyu0603@163.com (H.L.); yundongliu413@163.com (Y.L.); mxj@stumail.ysu.edu.cn (X.M.)

**Keywords:** surface plasmon resonance, Ag-Cu films, high-sensitivity, refractive index sensor

## Abstract

A fiber/Ag-Cu films surface plasmon resonance (SPR) refractive index (RI) sensor composed of multimode fiber-no-core-fiber-multimode fiber (MMF-NCF-MMF) structure is designed. The sensing region length and Cu film deposition time of sensor are gradually optimized by the control variable method, which finally achieves the improvement of sensor properties. We experimentally compared the sensing performance of the fiber/Ag film and fiber/Ag-Cu films sensor. Experimental results show that the fiber/Ag-Cu films sensor has good linearity (R-square = 0.993), and its sensitivity is as high as 3957 nm/RIU in the refractive index detection range of 1.3328–1.3853, which is 1109 nm/RIU higher than the sensitivity of a conventional fiber/Ag film sensor. The sensor presented in this paper adopts the structure with composite metal film, which outperforms the common single-layer metal film in chemical stability such as oxidation resistance and mechanical hardness. Meanwhile, the SPR sensor with MMF-NCF-MMF structure has the advantages of convenient manufacture and compact structure. In conclusion, it can bestow a unique advantage in the field of biological detection or chemical analysis.

## 1. Introduction

With the rapid development of technology and the continuous improvement of people’s quality of life, the demand for high-performance and easy to integrate sensors is increasingly urgent. Optical sensors are non-invasive and offer high-speed response times, which makes them increasingly popular for practical applications. For example, the design of optical sensors based on dielectric materials has opened up an entirely new exploration direction for optical sensing in recent years. This all-dielectric optical sensing method provides a potential application prospect for the new generation of sensors [1,2]. At the same time, surface enhancement techniques based on surface plasmon resonance (SPR) effects have been flourishing. The plasmonics research field has been recognized as a motivating principle for the development of advanced and practical optical sensor devices, which have brought outstanding results such as Terahertz plasmonic sensors [3], SPR and localized surface plasmon resonance (LSPR) sensors [4], and SPR optical sensors [5,6,7,8]. SPR optical sensors have been widely used for their superior features of real-time monitoring, high sensitivity, and label-free [9]. Especially with the development of photonic crystal fiber (PCF) technology, a new platform has been provided for the application of SPR in optical sensing. Various types of SPR-based PCF sensors have been manufactured with the advantages of simple structure, high accuracy, and small size, which further broadens the application prospect of SPR fiber sensors [10,11,12]. However, as the requirements for detection accuracy of various detection technologies are gradually increasing, how to further improve the performance has become a major challenge in the field of SPR optical sensors.

To overcome this challenge, a conventional processing method is to change the symmetric structure of fiber using micro-operating platforms, such as the proposed U-type fiber sensors [13] and etched fiber sensors [14]. Zhang et al. [15] presented a U-shaped curved fiber SPR sensor with a graphene/AgNPs structure by utilizing the advantages of combining several materials. By changing the laser-induced time, the sensor achieved the optimal time for deposition of AgNPs. With the proposed graphene/AgNPs U-bent fiber optic sensor, observed respectively for the detection of 90% aqueous ethanol and 20% aqueous glucose. These experimental results indicate that refractive index sensitivity of the proposed U-bent fiber sensor is 1198 nm/RIU. Ben et al. [16] proposed a sensor based on a cladding-etched thin-core single-mode fiber (TCSMF) sandwiched between two single-mode fibers. The cladding of the TCSMF is etched to enhance the leakage intensity of the evanescent field, which increases the sensitivity of the sensor. The experimental comparison proves that the refractive index sensitivity of etching increased at least 6 times to 857.5 nm/RIU in the RI range of 1.333 to 1.340. Moreover, with the decrease of cladding diameter of the TCSMF, the sensor sensitivity can be expected to be higher. Zainuddin et al. [17] proposed a sensor made of single-mode fiber modified by side-polishing method, which is based on the principle of SPR. The SPR dip can be shifted to a longer wavelength by controlling the cladding thickness on side-polished fibers. It was demonstrated that the side-polishing cladding SPR sensor had a higher sensitivity compared to the untreated sensor with a sensitivity measurement of 2166.667 nm/RIU. Because of the complexity of the above operation to improve sensor performance by changing fiber symmetry structure, a new method to improve performance has become a hot topic of current research, which is directly adding coating layer outside the fiber. Wang et al. [18] presented an Au film/barium titanate (BaTiO_3_) thin film SPR sensor. Compared with the traditional Au film SPR sensor, the sensitivity of the sensor is significantly improved, which is realized by charge transfer between Au and BaTiO_3_. The experimental results show that the sensitivity of fiber/Au film/BaTiO_3_ film SPR sensor is 2543 nm/RIU in refractive index range of 1.3332–1.3710, which is 495 nm/RIU higher than that of traditional Au film SPR sensor. Niu et al. [19] proposed a D-type large-core fiber sensor based on the coupling of Au film with Au nanoparticles. The simulation results show that the gap coupling electric field intensity between Au nanoparticles and Au film is 4–5 times higher than that of Au film, and the electric field intensity on the surface of Au nanoparticles is twice that of Au film. The refractive index sensitivity of the sensor was improved through exploiting the electric field coupling effect between Au film SPR and Au nanoparticles localized surface plasmon resonance (LSPR). The refractive index sensitivity of D-type fiber/Au film/Au nanoparticles sensor was demonstrated to be 3074 nm/RIU, which is 1.4 times higher than that of D-type fiber/Au film sensor. The SPR-LSPR coupling effect used in this design is a good method to enhance the sensing performance of optical fiber sensor, particularly to detect the change of refractive index of surrounding. Although sensor sensitivity can be effectively improved by the above method, the manufacturing process is complicated. At the same time, the fiber becomes very fragile due to the mechanical strength of fiber being damaged in the process of sensor manufacturing, which is not conducive to subsequent practical applications and preservation [20].

Based on this scientific issue, we proposed a high-sensitivity SPR refractive index sensor based on no-core fiber with Ag-Cu composite films. The Ag film is adhered to the NCF surface by silver mirror reaction, then coated with Cu film outside the silver film through the bronze mirror reaction, which modifies the position of resonance wavelength and prevents the silver layer from oxidation simultaneously. We first separately investigated the effects of different analytes, sensing region length and bronze mirror reaction time on sensor performance using the control variable method. Through the experimental results of this research, it is confirmed that the analyte is glucose solution, the length of sensing region is 2.0 cm and the copper mirror reaction time is 15 min. Finally, the fiber/Ag-Cu films sensor has been fabricated and its performance has been compared with that of conventional fiber/Ag film sensor through sensing experiments. The experimental results show that the sensitivity of fiber/Ag-Cu films SPR sensor is 3957 nm/RIU in the refractive index range of 1.3328–1.3853, which is 1109 nm/RIU higher than that of the conventional Ag film SPR sensor. The sensor has broad application prospects in the field of refractive index detection for industrial production and environmental monitoring.

## 2. Experimental Principle

SPR is generated by the excitation of surface plasmon polaritons, which are produced by a combination of electromagnetic waves and free electron density oscillations at the surface of the metal and dielectric medium film. When the light propagates from MMF to NCF, the incident light wave that can reach the interface between the metal and medium is called the evanescent wave. The wave vector component of this evanescent wave at interface is:

(1) $$K_{z}=\frac{\omega }{c}\sqrt{\varepsilon_{0}}sin\theta$$

where *ω* and *θ* express the angular frequency and the incidence angle of incident light, respectively. *c* refers to the speed of light, and *ε*_0_ represents the dielectric constant of cladding.

**Figure 1:**
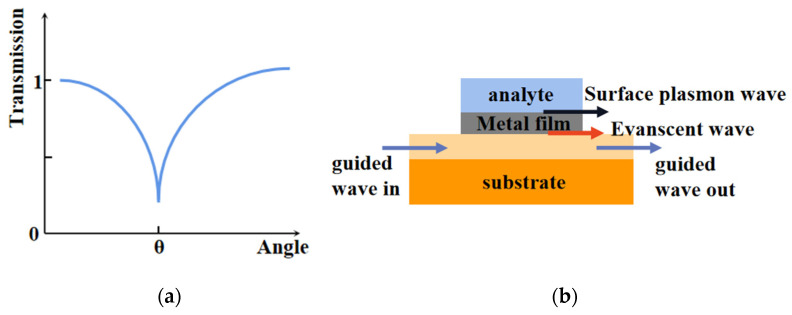
(**a**) Schematic diagram of SPR effect of double-layer metal film; (**b**) Model diagram of double-layer metal coating.

The SPR curve diagram of double-layer metal film is given in Figure 1a, when different kinds of metals are coated on the outside of fiber, the different effective refractive index of metal will lead to different phase matching points. At this time, the light intensity reflection curve produces blue peaks at the position of *θ*. We deposit bilayer metal film according to the model in Figure 1b, where the propagation of light depends on total internal reflection. Partially transmitted light exists as an evanescent wave when the light is transmitted in waveguide-coupled devices. Under resonance conditions, an evanescent wave will pass through the metal film, which will be influenced by analyte. The wave vector of surface plasma wave (*SPW*) excited at the cladding/metal interface is given by:

(2) $$K_{spw}=Re\left[\frac{\omega }{c}\sqrt{\frac{\varepsilon_{1}\varepsilon_{2}}{\varepsilon_{1}+\varepsilon_{2}}}\right]$$

Here, *ε*_1_ and *ε*_2_ are the dielectric constants of metal and external medium to be measured respectively, while *ε*_1_ varies with the light wavelength.

When the phase-matching condition is satisfied, the electromagnetic fields of evanescent wave and SPW are strongly coupled together, which results in part of the incident light energy being absorbed rapidly. As a consequence, the intensity of reflected light decreases, and a significant loss dip appears in transmission spectrum. The dip wavelength is called resonance wavelength, correspondingly. Since SPW is restricted to propagating near metal film, the resonance wavelength is very responsive to RI change of the external analyte to be measured. Therefore, when the RI of analyte changes, the phase matching conditions change simultaneously, which leads to the shift in position of resonance wavelength. Thus, the relationship between RI of analyte and the resonance wavelength can be determined by monitoring the shift of resonance wavelength in absorption valley, which further achieves the measurement of analyte RI. Sensitivity is one of the primary parameters reflecting sensor performance, which is defined as the ratio of the change in resonance wavelength (∆*λ_peak_*) to the change in adjacent refractive index of medium to be measured (∆*n_a_*). It can be represented as [21]:

(3) $$S_{\lambda }=\frac{\Delta \lambda_{peak}}{\Delta n_{a}}nm/RIU$$

## 3. Experiments

### 3.1. Fabrication of Sensor

Through the elaboration of experimental principles, the following sensing experiments were carried out, which can facilitate understanding of the resulting sensitivity. In this experiment, no-core fiber is used for the sensing region, which is easy to leak energy from inside to outside because of its simple structure. When metal film is deposited on the fiber surface, the metal film reacts readily with energy leaking from the core region. The parameter of experimentally used no-core fiber is measured by optical microscopy, where the fiber diameter is about 130.2 μm. The core and cladding diameters of MMF are 62.5 μm and 125 μm.

**Figure 2:**
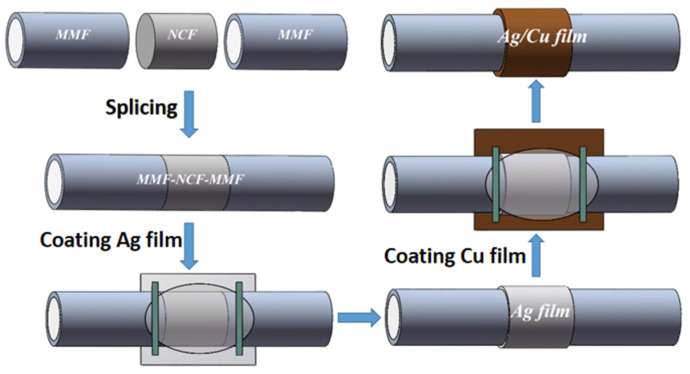
Fabrication process of MMF-NCF-MMF SPR sensor probe.

**Figure 3:**
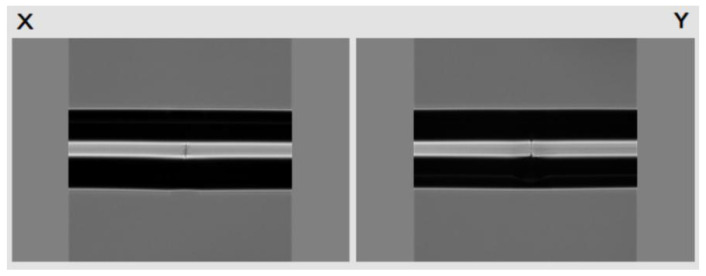
The alignment fusion of MMF-NCF-MMF.

**Figure 4:**
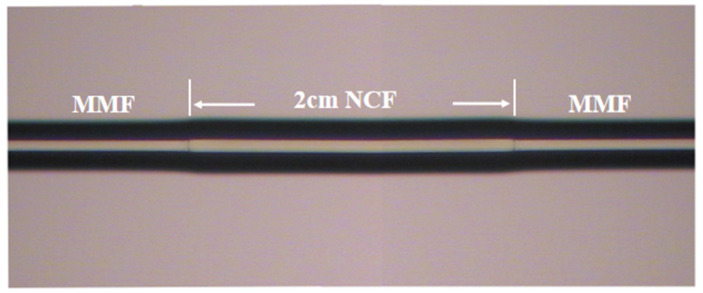
Microscopic image of the MMF-NCF-MMF.

**Figure 5:**
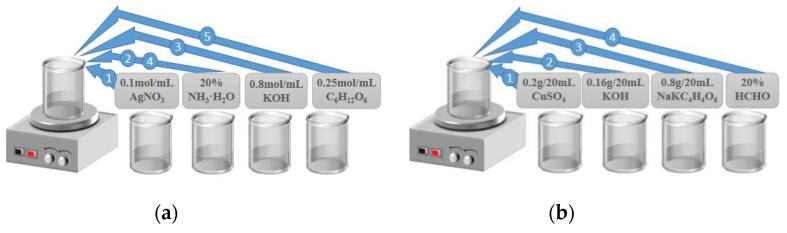
(**a**) Preparation process of reagent required for silver mirror reaction; (**b**) Preparation process of reagent required for bronze mirror reaction.

The manufacturing and modification process of fiber/Ag-Cu films sensor probe is depicted in Figure 2. First, both ends of the 2 cm-long NCF were fused to MMF jumper with well-cut end faces by the fusion splicer (Type-81CFA, Japan). The alignment fusion process of MMF jumper to NCF is demonstrated in Figure 3. Since the diameter of NCF selected in experiment is close to that of MMF, the automatic fusion mode can be used for fiber fusion operation. This considerably saves the workload and reduces the waste of fiber caused by optimizing setting. After both ends of NCF are fused to MMF jumper respectively, the MMF-NCF-MMF structure required for sensing probe is obtained, while the microscopic image is displayed in Figure 4. In order to ensure the uniformity of the coating, the fused MMF-NCF-MMF fiber structure was suspended and fixed on the glass plate. Next, preparation procedures of the two reagents required for metal coating were introduced, which are depicted in Figure 5a,b, respectively. We added silver nitrate solution (0.l mol/L) to the beaker placed on magnetic stirrer, then turned on the magnetic stirrer and slowly drop ammonia water with a volume concentration of 20% into the silver nitrate solution. Then we observeed the color of solution while dropping ammonia until the solution in beaker changes from brown to transparent. Next, potassium hydroxide (0.8 mol/L) was added to the above solution, stirring slowly while dropping ammonia water until the precipitate just dissolves, and the clarified reagent was silver ammonia solution. Silver ammonia solution was mixed with glucose solution (0.25 mol/L) in the ratio of 1:4, and then transferred to the sensor probe site after rapid stirring. After 20 min waiting for solution reaction, the residual solution was rinsed off with deionized water. After the above steps, the deposition of Ag film on MMF-NCF-MMF fiber structure exterior has been realized. Subsequently, copper sulfate solution (0.2 g/20 mL) and potassium hydroxide solution (0.16 g/20 mL) were injected into a beaker on a magnetic stirrer to prepare the bronze mirror reaction reagent. When the blue flocculent precipitate was observed in the beaker, an equal amount of potassium sodium tartrate solution (0.8 g/20 mL) was added to complete the preparation of Fehling’s reagen. Finally, we added 20% volume fraction of formaldehyde solution with strong reducibility to the Fehling’s reagent, at which time bronze mirror reaction reagent configuration was completed. Bronze mirror reaction reagent is uniformly covered for 15 min on NCF sensing site which has been coated with silver layer, and the probe was cleaned with deionized water after the reaction was completed. Bronze mirror reaction reagent is covered on NCF sensing part which has been coated with a silver layer and allowed to react for 15 min. We removed the sensor after the reaction, rinsed it with deionized water and dried it naturally at room temperature. After the above steps, fiber/Ag-Cu films SPR sensor manufacturing is completed.

### 3.2. Experimental Setup

**Figure 6:**
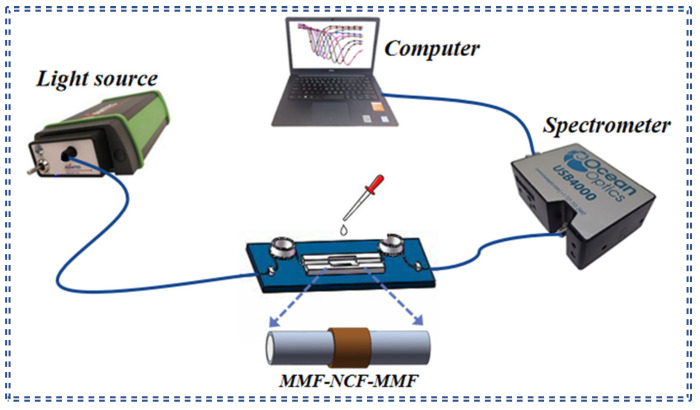
Schematic diagram of the experimental setup.

In order to explore sensing characteristics of fiber/Ag-Cu films SPR sensor, the experimental detection system displayed in Figure 6 was used. The detection system consists of a halogen tungsten light source (AvaLight-HAL-Mini, Beijing, China) with the working wavelength range of 400–2500 nm, a fusion MMF-NCF-MMF fiber structure, a spectrometer (Ocean Optics USB 4000, Beijing, China), and a computer. The refractive index of analyte was measured by an Abbe refractometer. In experimental preparation, the sensor was connected with halogen tungsten lamp and spectrometer by jump line while the sensor was fixed horizontally on the glass plate. Light source and spectrum analysis software were opened for experimental operation, and the whole measurement process was performed at room temperature. The light from halogen tungsten lamp enters the core of MMF at one end and is subsequently transmitted to NCF, which is entirely immersed in analysis fluid to be measured, and then input through NCF to MMF at the other end. A spectrometer combined with a computer is responsible for receiving and recording transmission signal. Firstly, the transmission spectrum of the sensor in air was recorded as the reference spectrum, and then the analytes with different refractive indexes were added to observe and record the position change of the resonance spectrum. After each time period, the analyte with different refractive indices is measured, it is essential to rinse the sensing area with deionized water in order to remove residual liquid from the previous time period. The refractive index sensitivity of the sensor is calculated by processing the data after completing the refractive index real-time sensing experiment.

## 4. Results and Discussions

### 4.1. Selection of Experimental Analyte 

**Figure 7:**
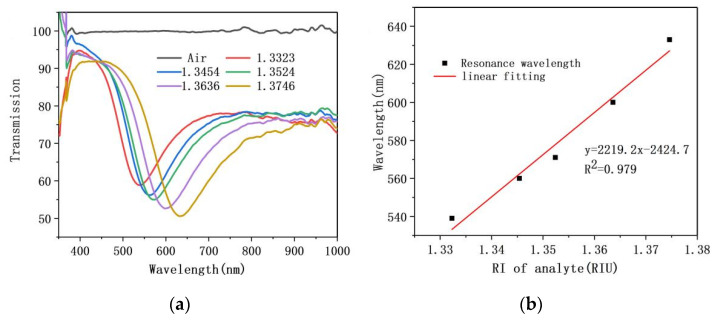
(**a**) The resonance spectrum of fiber/Ag film SPR sensor with sodium chloride solution as analyte; (**b**) The sensitivity fitting curve.

The selection of suitable analyte is an essential step before the sensor sensing experiment. In this section, sensing experiments were carried out with sodium chloride solution and glucose solution respectively, and appropriate analyte was selected by analysis of the results. The fiber of the sensing part is 1.5 cm NCF coated with a silver film single layer (silver film deposition time is 20 min). When analyte adopts sodium chloride solution with a refractive index range of 1.33–1.37, as illustrated in Figure 7a, the transmission valley has a red-shifted and valley depth deepens with the increasing refractive index of solution. The fitted curve of refractive index and resonance wavelength of sodium chloride solution is given by Figure 7b, with the fitted curve equation as:

y = 2219.2x − 2424.7

Here, x and y denote the refractive index of sodium chloride solution and the resonance wavelength of SPR dip, respectively. It can be seen from equation that the sensitivity of fiber/Ag film SPR sensor is 2219.2 nm/RIU when sodium chloride solution selected as the analyte.

**Figure 8:**
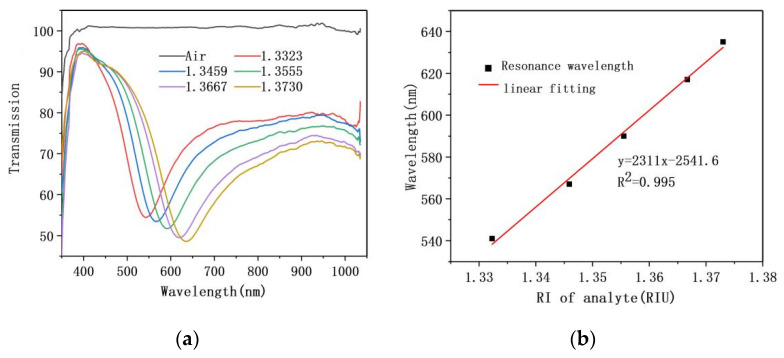
(**a**) The resonance spectrum of fiber/Ag film SPR sensor with glucose solution as analyte; (**b**) The sensitivity fitting curve.

After the experiment, the sensor was rinsed off using deionized water. On the premise of keeping other experimental conditions unchanged, the analyte was replaced by glucose solution with the same refractive index of 1.33–1.37 for sensing detection again. As shown in Figure 8a, due to the increasing refractive index of glucose solution, the transmission valley moves toward the long wavelength direction while the depth gradually deepens, which is similar to the experimental phenomenon of sodium chloride solution. When the sensor uses glucose solution as the analyte, the linear fitting relationship between the refractive index of analyte and resonance wavelength is indicated in Figure 8b, where the formula is given as follows:

y = 2311x − 2541.6

It is known from equation that when glucose solution is selected as analyte, the sensitivity of fiber/Ag film SPR sensor is 2311 nm/RIU. The results of two experiments groups proved that the sensor exhibited good stability when using different analytes. In order to prevent the reaction between sodium chloride solution and silver ion on the surface of sensor from affecting experimental results, we decided to choose glucose solution as an analyte for the next experiment.

### 4.2. Selection of No-Core Fiber Length

**Figure 9:**
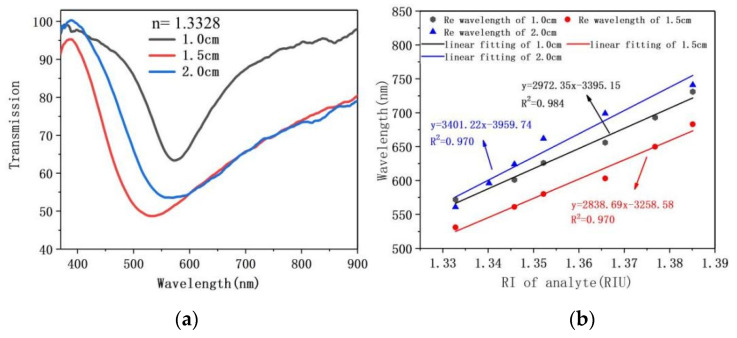
(**a**) Transmission spectrum of fiber/Ag film sensor at a refractive index of 1.3328 under different lengths of NCF (in cm); (**b**) Sensitivity fitting curve of fiber/Ag film sensor under different NCF lengths (in cm).

In this section, we separately selected three different lengths of 1.0 cm, 1.5 cm, and 2.0 cm solid-core NCF coated with single silver film (silver film deposition time is 20 min) to fuse with MMF for the sensing test. Then, three groups of fiber/Ag film SPR sensors with different lengths were placed in glucose solution that has a refractive index of 1.3328 for sensing experiments, respectively. The transmission spectrum and sensitivity fitting curves of different length sensors are obtained, which are revealed in Figure 9a,b. It was concluded from Figure 9a that as length increased from 1.0 cm to 2.0 cm, the depth of SPR exhibited a first increasing and then decreasing trend, where the positions of three resonance wavelengths were located at 572.77 nm, 532.77 nm and 562.13 nm, respectively. The sensitivity fitting curve of NCF with different length in Figure 9b shows that sensitivity improved from 2972 nm/RIU to 3401 nm/RIU as the increase of length from 1.0 cm to 2.0 cm, and the linearity changes slightly. Therefore, comparing the depth of SPR dip, the narrow full width at half maximum (FWHM) and sensitivity, we choose the NCF with the length of 2.0 cm as the optimal length for the following MMF-NCF-MMF sensing structure.

### 4.3. RI Measurement Experiments with Fiber/Ag Film Sensor

**Figure 10:**
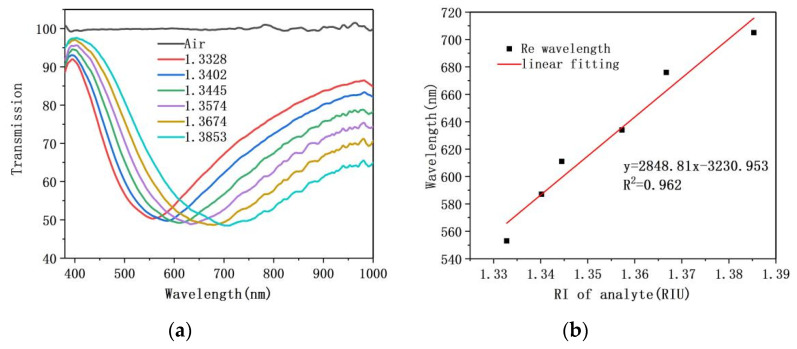
(**a**) Resonance spectrum of fiber/Ag film SPR sensor as the refractive index of the analyte increases; (**b**) The sensitivity fitting curve.

On the premise of determining analyte and sensing region length, the sensing characteristics of fiber/Ag film SPR sensor and fiber/Ag-Cu films SPR sensor were comparatively studied, and the proposed bilayer film sensor sensitivity enhancement effect was verified. Fiber/Ag film SPR sensor’s refractive index experimental resonance spectrum and sensitivity fitting curves are presented in Figure 10a,b. It is noticeable from Figure 10a that the position of SPR dip shifts toward long-wavelength as the refractive index of glucose solution increases from 1.3328 to 1.3853. The expression between the refractive index of glucose solution and the position of resonance wavelength is written as follows:

y = 2848.81x − 3230.953

where x and y denote the refractive index of glucose solution and the resonance wavelength of SPR dip, respectively. It is indicated from Figure 10b that the sensitivity of fiber/Ag film SPR sensor is 2848.81 nm/RIU and linearity is 0.962.

### 4.4. Time of Bronze Mirror Reaction

**Figure 11:**
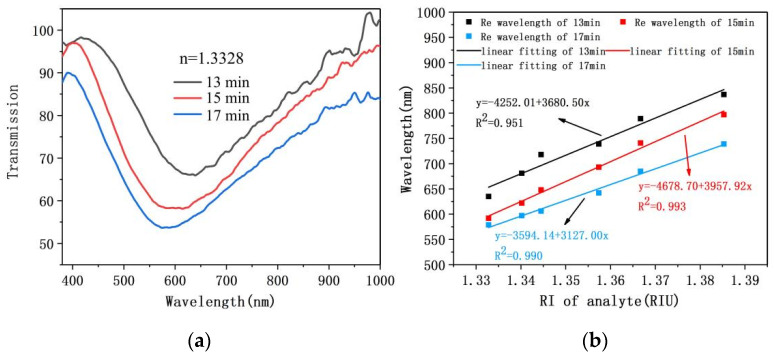
(**a**) Transmission spectrum of fiber/Ag-Cu films sensor at a refractive index of 1.3328 under different bronze mirror reaction time (in minutes); (**b**) Sensitivity fitting curve of fiber/Ag-Cu films sensor under different bronze mirror reaction time (in minutes).

The thickness of Cu film on fiber/Ag-Cu films SPR sensor is influenced by reagent concentration and reaction time. Based on the early running-in of the reagent concentration, this work only discusses the effect of reaction time on transmission spectrum. Figure 11a shows that the transmission spectrum of fiber/Ag-Cu films SPR sensor under the condition of analyte refractive index is 1.3328 and the bronze mirror reaction times are 13 min, 15 min and 17 min, respectively. It can be clearly seen in Figure 11a that SPR dip depth shows the gradually increasing trend as reaction time increases from 13 min to 17 min. Three SPR dips resonance wavelength positions were 628.932 nm, 593.028 nm, and 577.337 nm for the reaction times of 13, 15, and 17 min, respectively. This is because when the reaction time is increased from 13 to 17 min, the increase in Cu film thickness will excite more surface plasma. Moreover, when deposition time was increased to 17 min, Cu film became too thick for the electric field to penetrate easily, resulting in the SPR phenomenon being attenuated. The influence of different deposition times on sensor characteristics can be more clearly visualized in Figure 11b. Fiber/Ag-Cu films SPR sensor with three different Cu film deposition time was separately tested for RI sensing in the refractive index range of 1.3328–1.3853. The experimentally measured relationship between the position of resonance wavelength and the refractive index of glucose solution was depicted by linear fit in Figure 11b. By comparison, it was found that comprehensive performance of sensor was superior under the condition of 15 min reaction time, with the sensitivity and linearity reaching 3957.92 nm/RIU and 0.993, respectively. Considering the strength of SPR, the shape of transmission spectrum and sensitivity, 15 min was finally chosen as the optimal time for bronze mirror reaction.

### 4.5. RI Measurement Experiments with Fiber/Ag-Cu Films Sensor

**Figure 12:**
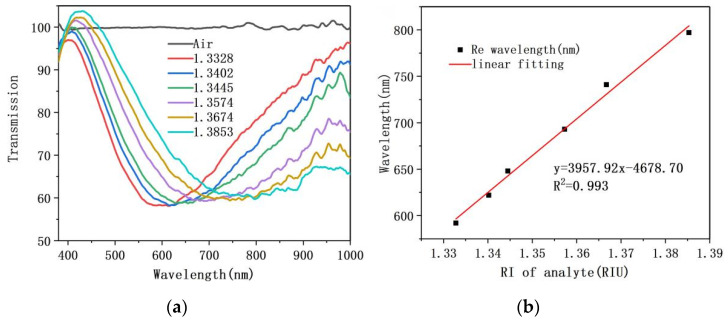
(**a**) Resonance spectrum of fiber/Ag-Cu films SPR sensor as the refractive index of the analyte increases; (**b**) The sensitivity fitting curve.

Similarly, RI sensitivity of the sensor with the fiber/Ag-Cu films structure was also investigated. The RI range of glucose solution used in the experiment is 1.3328–1.3853, which is consistent with previous experiments. Transmission spectrum and sensitivity fitting curve of this sensor were obtained, as shown in Figure 12a,b. From Figure 12a, as the RI of glucose solution increases from 1.3328 to 1.3853, the position of sensor transmission valley shifts toward the long wavelength. Compared with the transmission spectrum of the fiber/Ag film sensor, the transmission Valley depth of this sensor is shallower. This is because of the increased overall thickness with metal film, which makes it difficult for the electric field to penetrate, and eventually leads to the weakened strength of SPR. As Figure 12b illustrates, the experimentally measured RI sensitivity of the fiber/Ag-Cu films sensor was 3957 nm/RIU with a linearity of 0.993. It can be concluded that the double-layer metal film further improves the RI sensitivity of sensor by about 1109 nm/RIU compared to single-layer Ag film. As presented in Table 1, the performances of our proposed two sensors are compared in more detail. Summarizing the above experiments, it is concluded that, although the double-layer metal film increases the difficulty of electric field penetrating metal film, it has a significant effect on improving performance of the sensor in terms of sensitivity, linearity, and other important indicators.

### 4.6. Comparison and Analysis

As shown in Table 2, compared with the existing fiber sensors in sensing principle, RI measurement range and sensitivity, the fiber/Ag-Cu films SPR sensor proposed in this paper has excellent performance.

## 5. Conclusions

A fiber/Ag-Cu films SPR sensor based on MMF-NCF-MMF structure was designed and fabricated to improve the sensitivity of the SPR sensor. Metallic silver and copper with excellent chemical properties were proposed as the thin film materials to improve sensor properties. Under the condition of fixed silver film thickness, the influence of different bronze mirror reaction time on detection performance of SPR sensor was studied experimentally. The experimental results showed that the fiber/Ag-Cu films sensor has higher detection sensitivity at the bronze mirror reaction time of 15 min, which is 1109 nm/RIU higher than fiber/Ag film sensor without Cu film modification. The refractive index sensitivity reached 3957 nm/RIU, which is 1.4 times that of the fiber/Ag film sensor. By comparing the experimental results of the two kinds of sensors that were designed, we further illustrate the rationality and feasibility of this study for improving the sensor performance [26,27,28]. Therefore, the sensor proposed in this paper has wide application prospects in bio-detection and industrial production [29].

Although the performance of the sensor proposed in this paper has been improved, there is still great room for improvement in sensitivity. In order to further optimize the sensor performance, it is necessary to continually investigate metal thin film with good sensing properties and other materials that have superior performance.

## Figures and Tables

**Table 1:** The detailed performance comparison of two sensors proposed in this paper.

Transmission Spectrum	Fiber/Ag Film Sensor	Fiber/Ag-Cu Films Sensor
Detection range	1.3328–1.3853	1.3328–1.3853
Resonance wavelength (nm)	548	586
Linearity	0.962	0.993
Sensitivity (nm/RIU)	2848	3957

**Table 2:** Comparison with the SPR refractive index fiber sensor reported in the experiment.

Ref.	Sensor	Fiber Type	Refractive Index Range	Sensitivity (nm/RIU)
[19]	SPF/Au/gold NPS	SPF	1.3332–1.3710	3074
[20]	MMF/Ag	MMF	1.3328–1.3990	3223
[22]	MMF/Au	MMF	1.333–1.3469	1557
[23]	POF/Au	POF	1.33–1.41	2533
[24]	SPF/Ag	SPF	1.32–1.34	1798
[25]	SPF/Ag	SPF	1.333–1.360	1518
This work	NCF/Ag	NCF	1.3328–1.3853	2848
This work	NCF/Ag/Cu	NCF	1.3328–1.3853	3957

## Data Availability

The data presented in this study are openly available.
